# Supplementary material for: Determinants for male involvement in family planning and contraception in Nakawa Division, Kampala, Uganda; An urban slum qualitative study
Source: PLOS Glob Public Health. 2024 May 31;4(5):e0003207. doi: 10.1371/journal.pgph.0003207 (PMC11142587; doi:10.1371/journal.pgph.0003207)
Supplement: S1 Checklist — (DOC) [file pgph.0003207.s001.doc]

STROBE Statement—Checklist of items that should be included in reports of ***cross-sectional studies***

|  | Item No | Recommendation |
| --- | --- | --- |
| **Title and abstract** | 1 | (*a*) Indicate the study’s design with a commonly used term in the title or the abstract  **A qualitative design was applied** |
|  |  | (*b*) Provide in the abstract an informative and balanced summary of what was done and what was found  **A qualitative study was conducted among sexually active men in Nakawa Division, Kampala. A purposive sample technique was utilized to recruit 40 men for a Focus Group Discussion (FGD), and a convenient sampling method was used to sample 2 critical informants for the interview.** **The study found that male involvement in contraceptive use is influenced by natural and perceived factors such as poverty, cultural beliefs, and limited knowledge.** |
| Introduction | | |
| Background/rationale | 2 | Explain the scientific background and rationale for the investigation being reported  **Current evidence shows that male involvement in family planning is crucial to women's contraceptive use decisions. This study explored the reasons for the low level of male involvement in Nakawa Division, Kampala, Uganda. Understanding the influence of males in family planning uptake is essential to address the unmet needs of family planning.** |
| Objectives | 3 | State specific objectives, including any prespecified hypotheses  **To explore reasons for the low level of male involvement in FP in Nakawa Division, Kampala, Uganda using a qualitative study technique.** |
| Methods | | |
| Study design | 4 | Present key elements of study design early in the paper  **A qualitative design was applied in recruiting males between 18-45 from four of the fourteen neighbourhood areas in Nakawa Division****. Purposive sampling was used to select ten (10) participants from each neighbourhood based on the eligibility criteria. Convenient sampling was used to select two key informants to provide expert opinions on male involvement in family planning.** |
| Setting | 5 | Describe the setting, locations, and relevant dates, including periods of recruitment, exposure, follow-up, and data collection  **The study was conducted in the Nakawa division, the largest of the city's five administrative divisions in the Kampala district. The division is located in the eastern part of Kampala city with coordinates 0°20'00.0"N, 32°37'00.0"E. It has 23 parishes and 648 villages**. |
| Participants | 6 | (*a*) Give the eligibility criteria, and the sources and methods of selection of participants  **The eligibility criteria included being a male within the age stipulated, sexually active, and consented to participate in the study. The exclusion criteria consisted of unwillingness to take part in the survey. We ensured diversity of the participant by including participants within different age groups, diverse professions, educational background, socioeconomic and sociocultural backgrounds.** |
| Variables | 7 | Clearly define all outcomes, exposures, predictors, potential confounders, and effect modifiers. Give diagnostic criteria, if applicable.  **Men ‘s knowledge of family planning and contraceptives**   - **Understanding of family planning** - **Knowledge of and dealing with side effects of contraceptives** - **Men’s inappropriate attitude towards family planning and contraceptive use.** - **Contraceptive use promotes promiscuity and extramarital affairs**   **Utilization of contraceptives**   - **Side effects** - **Poverty**   **Perception men’s roles in family planning,**   - **Poor attitude toward male involvement in Family Planning** - **Limited knowledge of the roles of men in Family Planning**   **Perception men’s roles in family planning,**   - **Poor attitude toward male involvement in Family Planning** - **Limited knowledge of the roles of men in Family Planning**   **Community factors for men's participation in family planning**   - **Community’s perception on men’s participation in family planning** - **Sociocultural and religious factors for men’s involvement in family planning** |
| Data sources/ measurement | 8* | For each variable of interest, give sources of data and details of methods of assessment (measurement). Describe comparability of assessment methods if there is more than one group  **The data source for the study were from study participants (primary data)** |
| Bias | 9 | Describe any efforts to address potential sources of bias  **The Local Council (LC) chairpersons were conducted two months before the data collection to mobilize eligible participants in their respective neighbourhood for the FGDs. One researcher and two research assistants visited the selected study sites within two weeks to provide the study information such as purpose, risk and significance to participants' and who to report their decision to either or not to participate in the study. The LC Chairmen required eligible participants to refer other participants to the study. This snowball sampling helped find participants whose experiences were relevant to the study. The choice of a new subject was guided by the aim and objectives of the study. For those who consented to participate, a conference call was organized to help identify a mutually agreed location, date and time suitable for the FGD.** |
| Study size | 10 | Explain how the study size was arrived at  **Males aged between 18-45 years from four of the fourteen neighbourhood areas in Nakawa Division, namely; Ntinda, Mutungo, Naguru and Kyambogo years between March and June 2019. Purposive sampling was used to select ten (10) participants from each neighbourhood based on the eligibility criteria. Convenient sampling was used to select two key informants to provide expert opinions on male involvement in family planning. One of the key informants was a health worker at the Kiswa Kampala Capital City Authority Health Centre III who had superior knowledge about contraceptives and provided FP services and counselling for not less than ten (10) years. The other expert was the Local village Council (LC) chairman, the political leaders and heads of small communities within the districts of Kampala, who also make decisions in their areas of jurisdiction**. |
| Quantitative variables | 11 | Explain how quantitative variables were handled in the analyses. If applicable, describe which groupings were chosen and why  **The study is a qualitative study and the data was handled as;**  **Data processing and analysis were done iteratively with data collection, which helped to identify emerging themes and subthemes that directed subsequent interviews and aided in identifying data saturation. After carefully listening to each interview by the first author, those conducted in English were transcribed, whilst those in the local dialect were translated to English before transcribing. Although the first author is bilingual, linguistic experts verified the translated transcripts to ensure accuracy. NVivo 12 ©, a qualitative data analysis software, was used to manage the data. All transcripts were coded and read and re-read all transcripts to ensure all the salient points were captured. Codes were derived from the data rather than using a previously conceived coding framework to avoid truncating emerging ideas. Then during discussions among all the authors, the coding framework was reviewed and discussed as applied to the dataset. Pseudonyms have been used in the excerpts to protect the identity of the participants, and quotes from participants will be used to support subthemes in reporting the findings of the study.** |
| Statistical methods | 12 | (*a*) Describe all statistical methods, including those used to control for confounding  **Not applicable** |
|  |  | (*b*) Describe any methods used to examine subgroups and interactions  **Not applicable** |
|  |  | (*c*) Explain how missing data were addressed  **Not applicable** |
|  |  | (*d*) If applicable, describe analytical methods taking account of sampling strategy  **Not applicable** |
|  |  | (*e*) Describe any sensitivity analyses  **Not applicable** |
| Results | | |
| Participants | 13* | (a) Report numbers of individuals at each stage of study—eg numbers potentially eligible, examined for eligibility, confirmed eligible, included in the study, completing follow-up, and analysed  **Forty participants who were divided into four (4) FGD, each comprising ten discussants took part in the Focus Group Discussions** |
|  |  | (b) Give reasons for non-participation at each stage  **Not applicable** |
|  |  | (c) Consider use of a flow diagram  **Not applicable** |
| Descriptive data | 14* | (a) Give characteristics of study participants (eg demographic, clinical, social) and information on exposures and potential confounders  **Males aged between 18-45 years from four of the fourteen neighbourhood areas in Nakawa Division, namely; Ntinda, Mutungo, Naguru and Kyambogo years between March and June 2019** |
|  |  | (b) Indicate number of participants with missing data for each variable of interest  **Not applicable** |
| Outcome data | 15* | Report numbers of outcome events or summary measures  **Poor male involvement in family planning** |
| Main results | 16 | (*a*) Give unadjusted estimates and, if applicable, confounder-adjusted estimates and their precision (eg, 95% confidence interval). Make clear which confounders were adjusted for and why they were included  **Not applicable** |
|  |  | (*b*) Report category boundaries when continuous variables were categorized  **Not applicable** |
|  |  | (*c*) If relevant, consider translating estimates of relative risk into absolute risk for a meaningful time period  **Not applicable** |
| Other analyses | 17 | Report other analyses done—eg analyses of subgroups and interactions, and sensitivity analyses  **Not applicable** |
| Discussion | | |
| Key results | 18 | Summarise key results with reference to study objectives  **The study explored the determinants of males' involvement in FP and contraception among males and found that FP and contraceptives knowledge, socioeconomic, perception of men’s role, health facility and health professional related factors impact men’s involvement in FP.** |
| Limitations | 19 | Discuss limitations of the study, taking into account sources of potential bias or imprecision. Discuss both direction and magnitude of any potential bias  **The study was limited to males and did not solicit the views of females. Therefore, some side effects of contraception asserted by our participants that their female partners suffered could not be verified. Again, the study was geographically limited to Nakawa Division, and therefore the use of the findings must be contextualized.** |
| Interpretation | 20 | Give a cautious overall interpretation of results considering objectives, limitations, multiplicity of analyses, results from similar studies, and other relevant evidence  **Male involvement in contraceptive use is influenced by natural and perceived factors such as poverty, cultural beliefs, and limited knowledge. However, due to their impact on women's decisions, men must actively engage in family planning and contraceptive use. Interventions such as education and hands-on use of family planning and contraceptive services should be strategically designed to eliminate the barriers to men's participation in use to harness the benefits of men's involvement.** |
| Generalisability | 21 | Discuss the generalisability (external validity) of the study results  **Participants were recruited from four of the fourteen neighbourhood areas in Nakawa Division, namely; Ntinda, Mutungo, Naguru and Kyambogo. We ensured diversity of the participant by including participants within different age groups, diverse professions, educational background, socioeconomic and sociocultural backgrounds. Therefore, the findings of the study may have similar characteristics of the male population of the Nakawa Division and somewhat the population of Uganda.** |
| Other information | | |
| Funding | 22 | Give the source of funding and the role of the funders for the present study and, if applicable, for the original study on which the present article is based  **The authors would like to declare that a small grant was received for this study from Pan African University Life and Earth Science Institute, including Health and Agriculture (PAULESI), which covered logistic expenses such as duplication of the study tools, data collection and transportation.** |

*Give information separately for exposed and unexposed groups.

**Note:** An Explanation and Elaboration article discusses each checklist item and gives methodological background and published examples of transparent reporting. The STROBE checklist is best used in conjunction with this article (freely available on the Web sites of PLoS Medicine at http://www.plosmedicine.org/, Annals of Internal Medicine at http://www.annals.org/, and Epidemiology at http://www.epidem.com/). Information on the STROBE Initiative is available at www.strobe-statement.org.
