# Supplementary material for: Determinants for male involvement in family planning and contraception in Nakawa Division, Kampala, Uganda; An urban slum qualitative study
Source: PLOS Glob Public Health. 2024 May 31;4(5):e0003207. doi: 10.1371/journal.pgph.0003207 (PMC11142587; doi:10.1371/journal.pgph.0003207)
Supplement: Attachment — Submitted filename: Cover letter and Responses_(PGPH-D-23-01275).pdf [file pgph.0003207.s002.pdf]

Department of Epidemiology and Biostatistics

Fred N. Binka School of Public Health

University of Health and Allied Sciences, Hohoe Campus, Ghana

07/12/2023

The Chief Editor

Plos One Global Health

Dear Editor,

<u>Submission of revised manuscript (PGPH-D-23-01275)</u>

I write on behalf of all authors to acknowledge the valuable time, comments and suggestions editors and reviewers for this Journal have contributed to our manuscript "**Determinants for Male Involvement in Family Planning in Nakawa Division, Kampala, Uganda; A Qualitative Study".**

Authors have acted on all major and minor suggestions made to by reviewers. All corrections have been track changed in red and green coloration for essay identification.

Attached is a table of responses to all comments made for your perusal and reference.

Once again, thank you very much for the opportunity to have our manuscript reviewed by your Journal.

Authors look forward to having positive feedback from you as soon as practicable and any other actions they maybe required of us.

Thank you.

Yours sincerely,

Corresponding Author

Table of responses to reviewers

Reviewer #1: This is a good topic and interesting results they can help dissuade beliefs that men are not interested. My comments are as follows.	Authors acknowledge the valuable comments and suggestions made by the Reviewer.
The conclusion in the abstract do not line up with the findings well enough, I would suggest not adding findings here but work with what your results found.	The abstract and the conclusion have been revised as suggested
Under data analysis, the sentence following the sentence with Nvivo 12 is awkward.	The statement has been revised and now reads<br>"The data was analyzed using thematic analysis"
The title for table 1 is awkward. It could be read as poor, like lower socio-economic, and also is it based on an assumption that these men are not involved? Do we know they are not?	The title of Table 1 has been revised and the table has been redesigned to reflect the themes and subthemes generated from the interviews in the manuscript.
Table 1 needs some editing, there is inconsistency in some formatting	The table has been redesigned to address the formatting inconsistency.

One quote is repeated almost verbatim, some women...it is on page 11 and also on page 13.<br>Also, the topic is repeated, when you report a result say everything you want to say about it, but then do not repeat it later.	Authors have taken note of the repetition and have revised accordingly by looking at the interviews again to replace the page 11 quote;<br><br>*"I understand some women experience nausea, weight gain, mood changes, breast tenderness, and changes in menstrual patterns when they take contraceptives but the side effects can also depend on the type of contraceptive method that the person takes."*
Page 15 first para, misspelled word.	"proving" has be spelled well as "providing"
The topic on page 17, women appreciating make involvement is not mentioned in the title or abstract on table of themes. I would either take it out or mention it earlier.	The heading "***The behaviour of health workers and service delivery***" is a subtheme under the main theme "**Health facility-related factors**" which has been started in Table 1. However, for the purpose of clarity, authors have deleted

	the heading ““***The behaviour of health workers and service delivery***” to have a continues flow under the “**Health facility-related factors**” heading.
The men's general statements about not supporting family planning are also not well captured in the title and abstract and table, so it’s not just why they aren't interested to be involved, but the study also confirms that most do not want women using contraceptives. I would capture that better early on. Men’s lack of support for FP and the reasons.	Authors used the word “determinant” in the title to refer to “factor or reasons that may influence male to involve themselves in FP. This captures the suggestion “Men’s lack of support for FP and the reasons” by the reviewer. However, revisions have been made to the manuscript to reflect concerns made.
ALso the second statement about health facilities support to men given by a health care facility worker seems a bit off topic, I think its fine to have a lot of different topics but you need to frame it from the beginning.	The “**Health facility-related factors”** considered the barriers and enablers existing at health facilities to support or otherwise discourage males involvement in FP. The statement by a KI showed that some facilities prioritize women who come with their male partners for FP services. This can be used as a enabler

	to encourage males to participate in FP services with their wives at health centres.<br><br>*"At our health centre, we encourage male involvement, especially in the family planning counselling sessions. We even give couples priority when providing services. Those women who come with their husbands will be attended to promptly" (Key Informant 1)*
I think health facility related factors, and the behaviors of of health workers and service delivery could be combined as one topic. And the two quotes about men being treated poorly could be put together.	The health facility related factors, and the behaviors of of health workers and service delivery are considered as one topic/theme
The two quotes around money could also be put together rather than stating it once, moving to another topic, and then coming back to it..	The two quotes about financial support have been put together.
12. The paragraph under community factors is hard to understand, is the	The paragraph has been revised to address the ambiguity.

community frowned upon? Or does the community frown upon the couple?	
13. I feel like the discussion does not capture the breath of the results in the same way that the introduction and title and themes do not. Either streamline the results, or capture them.	The background and discussion have been revised as suggested
14. I think both the discussion and the introduction could be more streamline, its good that all the distinct topics are brought up, but it circles some	The background and discussion have been revised as suggested
**REVIEW # 2**<br>This qualitative study describes the determinants for male involvement in family planning in Nakawa Division, Kampala, Uganda. Authors conducted 4 FGDs with 10 participants each and two key informant interviews and reported the results. Finally, some of the main concepts of qualitative analysis such as double coding, inductive/deductive analysis, and data saturation appear to be missing. For the next round of	Thank you for the valuable comments and suggestions. Authors have acted on them accordingly.

reviews, authors should also provide line number to make it easier to offer feedback. Authors need to make the following major and minor revisions to be considered for publication. I request the authors to proofread thoroughly and copyedit to avoid any mistakes. Several statements have been made that have no citations. I suggest even checking the apostrophe such as here in Table 1 (it should be men's not Men 's) and minor typos throughout the manuscript. Please do a full spell check of the manuscript as well.	
Major revisions<br><br>BACKGROUND<br><br>Page 5: Last paragraph is about past studies and why the current study is needed. I suggest the authors to not mix with prior evidence on why men's	The last paragraph has been revised and now reads as; "However, how these programs targeted at males may affect the experiences of women in FP use,

involvement in FP is low. What do authors mean by not a single qualitative study from Uganda has explored this topic? Please add qualifiers such as, to our knowledge, we are not aware of any publications that have explored this topic quantitatively.<br>o Further, what do authors mean by “no information on change processes for male program beneficiaries”; please rephrase for clarity.	either positively or negatively is not well understood. Appreciating how males influence women use of FP has broader implications for uptake and changes in FP service delivery in Uganda. Unfortunately, men in Uganda are not targeted by Sexual and Reproductive Health programmes, and some men still consider sexual and reproductive health exclusive to women and are poorly informed. The study therefore assesses the factors that may influence males to get involved in FP in Nakawa, Uganda.”
METHODS<br>How did the authors screen males for sexual activity? Did you assume all men in 18-45 years were sexually active?<br><br>Why did authors choose only 2 key informants?	To screen for sexually active individuals, participants were assessed of their sexual history and activities, number of sexual partners in the last 6-12 months, history of getting a woman pregnant and how consistent or frequent has condom been used in the last 6 to 12 months.<br><br>40 individuals were used in the FGDs, eliciting much responses from the

	interviews. Responses by the 2 key informants was not yielding new themes as was given during the FGDs. So, authors decided on only 2 key informants.
Were FGDs conducted on video conference or in-person?	The FGDs were conducted in person by the researchers in community-based venues that the participants preferred.
Data collection is missing several important elements:-<br>o What did the FGD guide cover in terms of themes and topics?	The FGD guide covered six (6) main themes, including; (1) sociodemographic characteristics (2) knowledge of FP and contraceptives (3) utilization of contraceptives and side effects (4) perception of men's roles in FP (5) health facility influence of FP and contraception and (6) community influence of males' participation in FP and contraception.
How did authors determine 4 FGDs with 10 participants each was sufficient? Or in	Saturation was met when no new information or additional information, themes, or insights emerged from the

other words, how define how saturation was achieved.	responses given by participants during the interviews. As recommended in a systematic review, between 4-8 FGDs are required to achieve data saturation.[31] Therefore, in this study we used 4 FGDs with 10 participants each to reach data saturation. Again, each participant (that is 40 individuals) was made to contribute to the discussions to gather sufficient amount of data to adequately address the research questions and objectives and further data collection is unlikely to yield substantially different findings or deepen the understanding of the phenomenon under study.
Please use either participants or discussants for consistency throughout.	Authors have replaced "discussants" with participants throughout for consistency
DATA ANALYSIS	

Please cite the NVivo 12 software properly.	The appropriate citation has been indicated.
How was the intercoder reliability established? How many researchers coded the data?	The inter-coder reliability was conducted by two of persons to measure the consistency or agreement independently by assessing the same data and content. This was done to achieve a high inter-coder reliability and to arrive at similar conclusions or categorizations from the data.
Check typo, it cannot be “will be used” in this sentence “Pseudonyms have been used in the excerpts to protect the identity of the participants, and quotes from participants will be used to support subthemes in reporting the findings of the study”.	The “will” has been changed to “was”
What is the SNW acronym?	SNW is an initial for an author.

The analysis section is missing whether the analysis approach – of coding, and whether deductive content analysis or inductive analysis was used.	Key data analysis approaches have been done in the manuscript
RESULTS<br><br>Although qualitative work does not require quantifying the results (for example, 10/15 perceived this…), we still need to know whether many participants, most participants, or few participants endorsed a theme or felt a particular way.<br><br>Re: the quote, please change the words from lame babies to something else. I understand it is verbatim but perhaps the English translation should slightly to	Adjectives such as "most" participants, have been used to qualitatively describe the endorsement by the study's participants.<br><br>The word "lame" has been defined in the manuscript as "disabled"

reflect the participants' sentiments. Check whether the participant means disabled babies? "Family planning is something a woman should take care of. I have also heard that women give birth to lame babies after discontinuing the use of family planning to get pregnant."	
Avoid using discussant, respondent, participant – and please use only of these words consistently and throughout the paper.	Participants has been employed throughout the study for consistency.
On Page 12: Under the utilization of contraceptives, most of the results appears to be only from FGD 2 and some from FGD 5– it is not clear why only one FGD is being used to highlight. Please add results from other FGDs as well. Is<br><br>FGD 5 a typo because my understanding is that there were only 4 FGDs in total?	The results have been updated to have responses from all the FGDs

Does FGD number indicate the respondent within a given FGD or FGD number (as in, FGD 1, FGD 2, etc.). Please clarify and edit as needed.	The FGD5 is a typo and has been corrected.
On Page 14, “were unwilling users of the artificial methods and preferred the natural method”, what do authors mean by “artificial methods”	Artificial contraceptive methods are various techniques and devices designed to prevent pregnancy by interfering with the process of conception.
Please edit the title “Perceptions of the role’s men play in family planning”	The title has been revised to “Perceptions towards males’ involvement in family planning”
What does this mean “The participants in this student disclosed that contraception related decisions”. It looks like the paper was written in a rush without sufficient proofreading/ editing.	The word “student” was a typo and has been revised to “study”.

It looks like the two themes "Health facility-related factors and behavior of health workers" could be combined into a single result.	The behaviour of health workers is subtheme under the "Health facility-related factors theme."
On pg 17. Under this "Community factors for men's participation in family planning", please edit this word-- The few actively engaged in family planning reported that the community was frowned upon by society.	The statement has been revised to read; "For participants who actively engaged in family planning reported that the community (or society) frowned upon its usage."
On page 18, edit "A key informant also revealed that some of their religions are completely against family planning, and therefore it prevents some men from being adequately involved; however, he asserted that religion is not always correct." Do authors mean some people of certain religious faith?	The statement has been revised and now reads;<br>"A key informant also revealed that some religions (faiths) are completely against family planning, and therefore, prevents some men from being adequately involved; however, the key informant asserted that religion is not always right about some of the beliefs and practices"

On Page 21, "Our study underscored that women whose partners supported their decisions and accompanied them to the FP clinic are happier than others who come to the service centres alone"<br>I don't remember seeing any theme speaking to this result. Since your study only interviewed men, I don't see how you can comment on women's feelings or perceptions.	There is a result with respect to this statement which has been indicated in the manuscript;<br>"Women are normally very happy to have their husbands participate. I see the ones who come with their husbands to the family planning clinic, and it gives me so much joy because we recommend male partner support at this health centre. The women whose partners do not participate are often unhappy when they narrate their ordeals. Some women resort to hiding contraceptive use because their partners do not approve and that saddens me too." (Key Informant 1).
Page 21. Similarly, this sentence "Others indicated that presence of their partners at the FP clinics adds the extra cost to FP services" – this is not clear. Who are	The partners here is used to refer to "males" who accompany their spouse to the FP clinics and have been clearly defined in the manuscript

the partners? The men or the women? It's very confusing as written.	
Page 22: "Religion has been a major factor determining individuals' uptake of FP services 46,47 and this was equally reported by our participants as a barrier to engaging in FP and contraceptive use." I did not see religion as a major deterrent to men's engagement in FP. None of the results except one quote from a key informant that mentioned religion. Please add religious factors in the results if religion showed up as a theme.	Religion was captured under the "Community factors for male's participation in family planning". Authors have added extra quote to the to religion.
"Indeed, there could be FP and contraceptive options that may not ran contrary to the religious beliefs of individuals." What does this statement mean? Edit for clarity.	The statement "Indeed, there could be FP and contraceptive options that may not ran contrary to the religious beliefs of individuals" meant that; not all instances will FP and contraception options affect or go against one's belief.
