# Supplementary material for: Determinants for male involvement in family planning and contraception in Nakawa Division, Kampala, Uganda; An urban slum qualitative study
Source: PLOS Glob Public Health. 2024 May 31;4(5):e0003207. doi: 10.1371/journal.pgph.0003207 (PMC11142587; doi:10.1371/journal.pgph.0003207)
Supplement: Attachment — Submitted filename: 2nd_Cover letter_RESPONSES_PGPH-D-23-01275.docx [file pgph.0003207.s003.docx]

University of Health and Allied Sciences

Fred N. Binka School of Public Health

Department of Epidemiology and Biostatistics

PMB 31

Hohoe Campus

15/03/2024

The Editor

PlosOne Global Health

Dear Sir/Madam,

**SUBMISSION OF REVISED MANUSCRIPT (PGPH-D-23-01275)**

Authors write to acknowledge the valuable comments and suggestions made towards our manuscript (PGPH-D-23-01275). Based on the suggestions made, authors have revised the title of the study to; “**Determinants for Male Involvement in Family Planning and Contraception in Nakawa Division, Kampala, Uganda; An Urban Slum Qualitative Study.”** This is important to reflect the study’s background, problem and setting.

Authors have tracked change (in red coloration) all corrections in the revised manuscripts based on the comments and suggestions made by the reviewers. Again, we have attached sample of the interview guides used to conduct the study as a supplementary file.

Once again, thank you for the opportunity to have our manuscript go through rigorous review. In the meanwhile, authors will wait for any further action that may be required of us in relation to our manuscript.

Kindly find the attached table for the responses made towards addressing the review comments.

Thank you.

Yours sincerely,

Corresponding Author

| COMMENTS | RESPONSE |
| --- | --- |
| Minor edits  On Page 14, “were unwilling users of the artificial methods and preferred the natural method”, what do authors mean by “artificial methods”. This should term “artificial methods” should be explained in a footnote in the manuscript because this is not a commonly used phrase in contraception/family planning. Authors replied to the comment by saying “Artificial contraceptive methods are various techniques and devices designed to prevent pregnancy by interfering with the process of conception.” Can you provide examples of such methods? I still don’t get it. Also, in addition to responding to reviewers, please add this info in the manuscript. | Thank you for the question and suggestion.  Authors have duly revised and included the meaning and examples of artificial methods of contraception. These include hormonal methods (oral contraceptive pills and Depo-Provera), the intrauterine device, barrier methods (the latex condom and the diaphragm), and sterilization (tubal ligation and vasectomy) (Pritchett and Fernández, 2001).  Pritchett, A.C. and Fernández, J.R. (2001) ‘Artificial Contraception is Associated with Increased Numbers of Induced Abortions’, *The Linacre Quarterly*, 68(4), pp. 348–357. Available at: https://doi.org/10.1080/20508549.2002.11877626. |
| On Page 23 “Others indicated that the presence of their male partners at the FP clinics adds an extra cost to FP services”. Why does presence of male partners add an extra cost to FP services? It is still not clear. | Generally, males have financially supported their female spouses to procure FP services. Some providers therefore leverage on the male presence to add other services and backdoor payments for the male spouse to pay. |
| Page 25: There is a line in the conclusion “I am tagging FP and contraceptive methods as women's business and misconceptions and myths about FP and contraceptives.” This does not make sense. Again, it seems the authors have not proofread and copyedited the manuscript sufficiently to ensure clarity in writing. Please spend sometime in proofreading and correcting small typos. | The “I am” has been deleted.  The manuscript has equally been proofread and edited. |
| Page 25: …replace this line and add “The health-related determinants for male involvement in family planning include an unfriendly family planning clinic environment, a lack of recognition of males as users of contraceptives, insufficient gender-focused discussions, inadequate information on men’s family planning methods, and unfriendly attitudes of health workers toward males in family planning”. Currently there are several typos in the line and it is grammatically incorrect. | The line has been deleted and typos corrected. |
| Reviewer #3: Overall, this is an interesting study that reveals insights into men’s participation or lack thereof in family planning in Uganda. The themes highlighted reflect notable findings and are sufficiently backed by the interview extracts. This has valuable implications to how FP interventions are delivered. The main critique here is that in the background literature, the authors emphasize the rural/urban differences in contraceptive use with rural areas having the highest number of children and least contraceptive use. Yet the study focuses on an urban population of men, and this should be included as a limitation of the study. Either the study title should adjust to reflect male participation in urban settings, or the study should include FGDs in a rural context to sufficiently explore the stipulated aim of the study.  The discussion should be strengthened by expanding on other studies that agree with or are contrary to the author’s findings. Additionally, the language in some sections comes across biased and needs to be rephrased. Further comments are included below.  Background (p.4) – Do men seldom involve themselves or have family planning initiatives historically excluded men and focused on women? It is worth acknowledging this aspect as well. | Thank you for the valuable comments.  The study area was an urban slum area. Authors have therefore revised the title to reflect the study setting. Similarly, the study background has been revised to be consistent with the title and study area.  The discussion has been strengthened as recommended.  Thank you for comments. Authors have revised as recommended. |
| Background (p.4) – “Evaluation of several early studies showed that men's involvement led to increased contraception uptake.” Include citations.  Background (p.4) – “The most frequent reason for the non-use of contraceptives is the lack of spousal support.” Include citations.  Background (p.4) - Men are the reason for most of the reproductive health issues suffered by their female partners in Uganda. This is a rather strong accusation and may lead to resistance. Rephrase. Is it the limited knowledge/culture that impacts their decision making and therefore how they influence family planning? | Authors have cited;   1. Sternberg P, Hubley J. Evaluating men’s involvement as a strategy in sexual and reproductive health promotion. *Health Promot Int*. 2004;19(3):389-396. doi:10.1093/heapro/dah312 2. Dumbaugh M, Tawiah-Agyemang C, Manu A, ten Asbroek GHA, Kirkwood B, Hill Z. Perceptions of, attitudes towards and barriers to male involvement in newborn care in rural Ghana, West Africa: A qualitative analysis. *BMC Pregnancy Childbirth*. 2014;14(1):1-9. doi:10.1186/1471-2393-14-269   Thank you for the comment. However, authors did not find any statement that reads “The most frequent reason for the non-use of contraceptives is the lack of spousal support.”  Thank you for the valuable comment. However, authors have not sought to suggest that men are responsible for reproductive health issues suffered by women and we are unable to locate any statement that seem to suggest such. The statement reads “In most developing countries, especially in the African communities, the decision-making process of a family is largely influenced by the males.”  Similarly, previous studies that have been cited showed that, limited knowledge of FP in males negatively impacts their involvement in FP use particularly in providing the needed support to their female partners. So addressing this as a challenge could help maximize their (males) support and increase FP service uptake. However, authors have rephrased/rearranged the paragraph for clarity. |
| Aim (p.5) – Are there specific questions broadly that were used to explore male involvement? If so, include them. Include interview questions in the appendix of the manuscript.  Study setting (p.6) – Why was Nakawa chosen as the study site? Relative to the question of low male involvement? Have SRH interventions been run in this area? Why not a rural site given the background information on women in rural areas likely having more children and less likely to use contraception than women in urban areas? Nakawa is considered urban. The rural-urban comparative would be considered critical in diversifying the participant pool.  Participant recruitment (p.7) – LC chairpersons were contacted not conducted.  Data collection (p.8) – FGD among males is a culturally sensitive topic and would have benefited from one-on-one interviews to illuminate more intimate perspectives and counter group think. Not conducting one on one interviews may need to be justified or be discussed as a potential study limitation. Include high-level examples of what types of questions were posed during the focus group discussions.  Data analysis (p.9) – it is not clear what data analysis method was employed. Is this thematic analysis? Content analysis? Clearly indicate inductive approach was taken to coding, if this is the case.  Findings (p. 11). Men’s inappropriate attitude – who judges it to be inappropriate. Rephase to men’s attitude or poor attitude towards FP and in the descriptions describe it and why it may be inappropriate. Otherwise again, this is alienating language.  Discussion p.19. It would be helpful to see literature that contradicts the study’s findings. All cited literature is consistent with the current study. Furthermore, for instance why is information on FP side effects not provided to patients based on the literature? You cite this accusation as true, why is it the case?  Discussion p.20. Take the time to identify at least what one of the citations you have included contributes to the issue of or intervention on side effects, myths or beliefs. What have some of the successful interventions looked like?  Discussion p.21. The claim around women being happy when escorted by husbands to service centres is made by one key informant’s perspective and did not come directly from the women. It is not accurate to make this claim on behalf of women unless validated by women participants.  Discussion p.22 Provide examples, beyond simply citing, of studies that show successful integration of culture/religion in FP initiatives to illuminate this point. Same applies to “an all-inclusive-approach”, what examples exist on this anywhere in the world. | Thank you for the suggestion. Authors have added the questions as a supplementary file.  Thank you for the valuable comments.  First, authors have given further details about the research problem in relation to why the Nakawa Division was chosen. The Nakawa Division is predominately a slum setting coupled several public health challenges. The current understanding of male involvement in FP and contraception is not known and therefore authors were motivated to address this gap.  The highlights of the rural and urban disparities of FP and contraception use was to give the background and magnitude of FP and contraception in Uganda. However, authors have revised the manuscript to be more focused.  Thank you for the correction. The word “conducted” has been deleted and replaced with “contacted”.  Thank you very for the comments and suggestion. Authors have attached a copy of the interview guide as an appendix. Again, authors have indicated in the manuscript, the design of the semi-structured interview guide under the Data Collection (Paragraph 2). Similarly, authors have added the interview guides used as a supplementary file.  The reason and limitations of the FGDs has been noted in the manuscript.  Thank you for comment. A thematic analysis was conducted.  Thank you for the comment.  The word “inappropriate” has been deleted and replaced with “poor”  Thank you for the suggestion. Authors have revised the manuscript and updated with other citations as suggested.  Thank you for the suggestion. Authors have revised the manuscript and updated with other citations where some interventions have been suggested, such as the us  Thank you for the suggestion. Authors have revised the manuscript to reflect the participants (KI) assertion and updated with other citations where male partners accompanied their female partners to family planning centres  Thank you for the suggestion. Authors have revised the manuscript and updated with other citations as suggested. The phrase “an all-inclusive-approach” was used to refer to using a couple-based approach to family planning. That is involving both males and females in family planning services. |
|  |  |
